# Neonatal single-site divided colostomy in anorectal malformation

**DOI:** 10.1007/s00383-026-06507-2

**Published:** 2026-06-29

**Authors:** Signe Olsbø, Astrid Ingeborg Austrheim, Anders Telle Hoel, Kristin Bjørnland

**Affiliations:** 1 Department of Hepatic, Pancreatic and Pediatric Surgery, Oslo University Hospital, Oslo, Norway; 2 Institute of Clinical Medicine, University of Oslo, Oslo, Norway; 3 Children’s Surgical Department, Division of Pediatric Surgery, Oslo University Hospital, Oslo, Norway

**Keywords:** Anorectal malformation, Neonatal colostomy, Stoma complications, Surgical outcomes

## Abstract

**Purpose:**

To evaluate outcomes of a single-site divided colostomy in neonates with anorectal malformations (ARM).

**Methods:**

A retrospective review was conducted of neonates with ARM undergoing colostomy creation between 2012 and 2024 at a tertiary referral center. The technique used a divided colostomy with the proximal limb raised ~ 1.5 cm above skin level and a narrowed distal mucous fistula within the same opening, allowing coverage with a single stoma bag. The distal bowel was irrigated at surgery. Complications were classified using the Clavien-Madadi (CM) system.

**Results:**

Sixty-one patients (79% male) were included, operated at a median age of 1 (1–3) day. Postoperative complications occurred in 12 (20%) patients. Two (3%) had CM IIIb complications (parastomal hernia and misidentification of bowel limbs). Urinary tract infection (CM II) developed in 6 (10%) patients; three with rectourethral fistula (one with vesicoureteral reflux), two with cloaca, and one without a fistula. Additional complications included granulation tissue requiring treatment (1) and wound infections requiring antibiotics (3). Major dressing difficulties were reported in 5 (8%) patients, while 20 (33%) had minor to moderate issues managed conservatively.

**Conclusion:**

Single-site divided colostomy is a safe technique with predominantly minor complications, minimal scarring, and no observed fecal overflow.

## Introduction

Anorectal malformations (ARM) comprise a spectrum of congenital anomalies affecting the rectum and anus. They occur in approximately 1 in 3000–5000 live births and typically require creation of a diverting colostomy in the neonatal period when no perineal or vestibular fistula is present [1]. Although colostomy creation is a well-established first step prior to definitive repair, the optimal stoma configuration remains debated.

To create a loop colostomy is technically easy and only requires a single opening. However, in ARM patients loop colostomies have been associated with fecal spillover into the distal limb, distal rectal contamination, and urinary tract infections (UTIs), particularly in patients with recto-urinary fistulas [2, 3]. Furthermore, prolapse rates up to 20% have been reported in some series [4, 5]. Therefore, many prefer a divided (double-barrel) colostomy creation in ARM neonates to reduce the risk of prolapse and distal contamination [2, 3]. However, this configuration traditionally requires two separated stomas divided by a skin bridge, which may increase the complexity of appliance fitting and daily stoma care. In addition, it results in a larger abdominal scar, which may have implications for body image and psychosocial well-being in patients with ARM [6, 7]. Lastly, peristomal skin complications are common in neonates, yet are rarely the focus in comparative studies of colostomy techniques, and detailed data on stoma care, skin integrity, and wound management are limited for the ARM population [8].

Variations of divided colostomies with both limbs brought through a single opening have occasionally been described, but detailed outcome data are limited [9]. At our institution, a divided colostomy with both limbs brought out through a single skin opening and with the distal bowel fashioned as a small mucous fistula has been the preferred method for the last two decades. To our knowledge, no studies have reported outcomes for this stoma technique in ARM neonates. The primary aim of this study was, therefore, to report our institutional experience and postoperative outcomes following neonatal single-site divided colostomy in patients with ARM. Secondary objectives were to assess the incidence of UTIs and to evaluate stoma-related, skin-related, and wound complications.

## Methods

### Study design

We conducted a retrospective chart review of neonates with ARM who underwent colostomy formation between January 2012 and December 2024. All patients were treated at Oslo University Hospital, a tertiary referral center for ARM in Norway. Patients were identified through an electronic search of the hospital’s medical records system. Inclusion criteria were a confirmed diagnosis of ARM and colostomy performed within the neonatal period, defined as the first 28 days of life. Clinical data were extracted from electronic medical records and included ARM subtype, associated anomalies, operative details, and postoperative complications.

### Surgical technique and follow-up

**Fig. 1:**
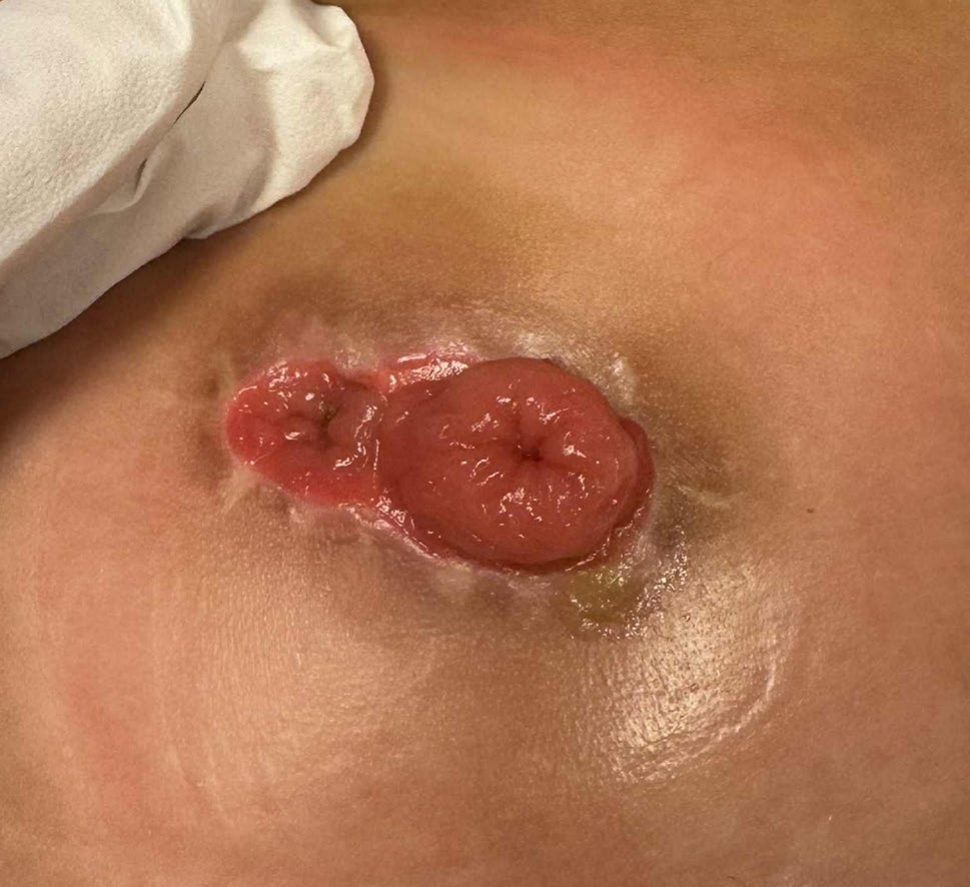
Postoperative appearance of the single-site divided colostomy several weeks after surgery in a patient with ARM. Used with permission from parents

Neonates without a visible perineal or vestibular fistula got a colostomy within 48 h after birth. The colostomy is created through a small transverse mini laparotomy in the left iliac fossa. First, the sigmoid colon is identified, and the proximal bowel is mobilized and delivered through the incision. The bowel segment selected for the proximal stoma is chosen to provide an appropriate tension-free position while avoiding excessive redundancy that could predispose to stomal prolapse. In most cases, this corresponded to the distal descending colon or proximal sigmoid colon. A colotomy is then made, and the distal bowel segment is vigorously irrigated with saline solution until clean. If the orientation of the bowel could not be determined with certainty, a catheter is inserted into the presumed distal segment, and a contrast study is performed to confirm its distal location. The colon is subsequently divided using monopolar electrocautery (cut mode) and brought through the rectus muscle withing the same skin incision (Fig. 1). The laparotomy incision is adjusted as necessary to ensure an optimal fit for the two bowel limbs. The proximal limb is sutured to the fascia with three absorbable braided sutures. If required, the fascial opening is narrowed with one or two absorbable sutures. The fistula is made by suturing the bowel to the skin with absorbable sutures flush to the skin and plicated to make it as narrow as possible. Lastly, the bowel wall of the proximal limb is everted by three 3-point tripod sutures and brooked to create a protruding spout approximately 1–1.5 cm above skin level. The bowel is secured to the skin with interrupted monofilament sutures, and an intracutaneous skin closure with quick absorbable monofilament suture is performed when necessary to reduce a too large skin opening.

All patients get one dose of prophylactic antibiotics before skin incision, and patients with recto-urethral fistula receive prophylactic antibiotics until definitive repair. As soon as the baby is awake, there are no feeding limitations. Postoperatively, parents receive structured education in stoma and skin care from dedicated stoma nurses. Families are followed closely through outpatient visits and telephone consultations. Stoma condition, skin integrity, and wound management are routinely documented in the medical records by the stoma nurses in separate clinical notes.

### Outcome measures

Postoperative complications were recorded and classified according to the Clavien-Madadi (CM) classification system, based on severity and the type of intervention required [10]. In addition, stoma/skin-related issues were assessed based on the documented need for local management and categorized into three groups: (1) No problem; (2) Minor to moderate problem (managed with sealing paste or advanced dressings); (3) Major problem (requiring more than two dressing changes per day). UTIs were recorded when clinically diagnosed and treated with antibiotics.

### Statistical analysis

Statistical analyses were performed using Stata version 18.0. Continuous variables are presented as means with standard deviations when normally distributed, and as medians with min-max when not normally distributed. Categorical variables are presented as frequencies and percentages.

### Ethics

The study was approved by the Hospital’s Data Protection Officer. Data was handled in accordance with national regulations and institutional guidelines for research using medical records.

## Results

### Cohort characteristics

A total of 70 patients were identified, of which 9 were excluded from the study due to having end stomas. Subsequently, 61 patients (79% males) were included. Urinary tract anomalies were present in 17 (28%) of the patients, renal agenesis being the most common (Table 1). Overall, three quarters of the patients exhibited other congenital anomalies. The median age at surgery was 1 (1–3) day. Intraoperative imaging to confirm proximal and distal loops was done in 9 (15%) operations. A consultant was the primary surgeon in 46 (73%) procedures. Median operative time was 76 (30–109) minutes. Median time from anoplasty to stoma closure was 9 (4–32) weeks.

**Table 1:** Demographic and clinical characteristics of patients with anorectal malformation (ARM) operated in the neonatal period with a single-site divided colostomy

Characteristics	Value
Patients (n)	61
Sex	
Male (n)	48 (78.7%)
Gestational age (mean, weeks)	38.6 (SD 2.4)
Types of ARM	
Urethral fistula (n)	41 (65.1%)
Without fistula (n)	14 (22.2%)
Cloaca (n)	6 (9.7%)
Urinary anomalies	
Renal agenesis (n)	7 (11.5%)
Vesicourethral reflux (n)	3 (4.9%)
Urethral duplication (n)	4 (6.5%)
Renal dysplasia (n)	1 (1.6%)
Renal fusion (n)	1 (1.6%)
Posterior urethral valves (n)	1 (1.6%)

### Postoperative complications

Postoperative complications occurred in 12 (20%) patients (Table 2). Two (3%) patients experienced CM IIIb complications: One patient developed a parastomal hernia 29 days postoperatively, and in another patient, the proximal and distal bowel limbs were switched. UTI (CM II) developed in 6 (10%) patients; three with rectourethral fistula (one with vesicoureteral reflux), two with cloaca, and one without a fistula (Table 3). Surgical site complications requiring antibiotics (CM II) occurred in 3 (5%) patients. No clinically apparent problems related to fecal overflow were recorded, and no fecal accumulation in the distal rectum was observed at the time of anoplasty.

**Table 2:** Post-operative complications following neonatal single-site colostomy in 61 patients with anorectal malformation

Complication	*N* (%)	Clavien–Madadi (CM) classification
Overall postoperative complications	12 (20)	
Parastomal hernia	1 (2)	IIIb
Switching of proximal and distal bowel limbs	1 (2)	IIIb
Urinary tract infections	6 (10)	II
Granulation tissue polyp requiring treatment	1 (2)	II
Surgical site infections requiring antibiotics	3 (5)	II
Stoma care-related problems		
Major dressing problems*	5 (8)	Not CM-classified
Minor to moderate problems**	20 (33)	Not CM-classified

*>2 daily dressing changes

**Treated with sealing paste or dressings

**Table 3:** Detailed description of six patients who developed a urinary tract infection after neonatal colostomy for anorectal malformation (ARM)

Gender	Type of ARM	Urinary anomaly	Other anomalies/comorbidities	Antibiotic prophylaxis
Male	Rectourinary fistula (rectoprostatic)	Renal agenesis	Caudal regression syndrome, omphalocele	Yes
Male	Rectourinary fistula (rectobulbar)	-	-	Yes
Male	Rectourinary fistula (rectobulbar)	Vesicourethral reflux grade IV, trabeculated bladder	Deformed sacrum, tethered cord	Yes
Female	Cloaca (common channel 2 cm)	Duplicated ureters	Caudal regression syndrome, absent vagina and uterus	Yes
Female	Cloaca (common channel 4 cm)	-	-	Yes
Male	No fistula	-	Down syndrome, bifid scrotum, hypospadias, congenital heart defect, ocular malformation	No

### Stoma- and skin problems

The majority (59%) of patients had no documented stoma related problems. Major dressing difficulties requiring more than two daily changes were seen in 5 (8%) patients. One of these required hospital admission due to a parastomal hernia (listed in Table 2). Minor to moderate problems were observed in 20 (33%) patients and were managed conservatively with adjustments in dressings or the use of sealing paste (Table 2). The minor problems consisted mainly of small leakages and skin irritation, managed by stoma nurses through outpatient visits or telephone advice. None required additional hospital admission.

## Discussion

This study demonstrates that a modified divided colostomy technique in neonates with ARM is a safe option leaving smaller scars than traditional divided colostomies, but still allows functional separation of the bowel limbs. The overall complication rate was 20%, the majority being grade CM II and managed conservatively. Colostomies in children with ARM are generally associated with a high complication rate. In a systematic review including 3000 patients with ARM or Hirschsprung disease, loop colostomies had higher overall complication rates than split colostomies (63% vs. 45%), mainly due to prolapse (18% vs. 6%), which was also the leading cause of stoma revision [2]. A large retrospective cohort including 589 patients with various indications for stoma formation reported an overall complication rate of 33%, with higher prolapse rates in loop stomas (16%) [11]. In another recent cohort of 276 children with ARM, the overall stoma-related complication rate was 24% [12]. Interestingly, the authors reported fewer complications in proximal (transverse) colostomies and in loop stomas compared with distal (descending or sigmoid) and split colostomies, which contrasts much of the earlier literature favoring distal divided colostomies [2]. However, because most proximal stomas in that cohort were loop colostomies, the independent effects of stoma type and location remain difficult to determine. In our series, no cases of prolapse were observed. We hypothesize that the afferent limb being at the junction between the descending and sigmoid colon and that the distal is sutured as a narrow fistula are important for the low prolapse rate. We had one case (2%) of switching of the proximal and distal limbs. This episode highlights that the correct tract should, as a rule, be verified, either radiological imaging or laparoscopy.

Distal contamination and fecal spill-over have been suggested as disadvantages of loop colostomies, particularly in patients with recto-urinary fistulas. Peña et al. reported high rates of UTIs in patients with loop colostomies and in split colostomies placed too close together [3]. However, most other studies report lower and less consistent UTI rates [2]. In the cohort reported by Klein et al., UTIs were more frequent in patients with split colostomies, although this was likely related to a higher prevalence of associated urogenital malformation rather than the stoma type itself [12]. In our cohort, UTIs occurred in 10% of patients and were observed in children with recto-urinary fistulas, urinary malformations or cloaca - conditions inherently associated with increased infection risk [13, 14]. One UTI occurred in a child without a fistula. The patient required postoperative urinary catheterization for fluid balance monitoring due to overhydration. Five days after colostomy creation, elevated inflammatory markers prompted antibiotic treatment for suspected urethral fistula. MCU showed neither urethral fistula nor vesicoureteral reflux. In the absence of rectourinary fistula or reflux, and given the prolonged catheterization, this episode was considered most likely catheter-associated, with urine culture findings supporting this interpretation. No clinically documented problems related to fecal overflow were recorded and no accumulation of feces was observed in the distal rectum during the anoplasties. As emphasized by Peña and others, rigorous irrigation of the distal bowel at the time of colostomy is mandatory to prevent retained stool and distal contamination [3].

Stoma- and skin-related problems were common, but predominantly minor. Peristomal skin complications are frequently reported in studies of neonatal colostomies, but are rarely described in detail [15, 16]. Reported rates vary widely, partly due to differences in definitions and reporting practices. For example, Ciğdem et al. reported skin excoriation in 47% of patients, although only 8% required hospital admission, suggesting that most cases were mild and self-limiting [4]. Similarly, van der Hondel et al. reported frequent, but generally mild skin excoriation [2]. In the study by Basuguy et al., excoriation and wound dehiscence were more frequent in split stomas with a skin bridge (14% each) than in loop stomas (9% and 8%, respectively) [11]. However, the cohort was not limited to neonates with ARM. In our cohort, most skin-related problems were managed conservatively, and severe cases were uncommon. Stoma- and skin-related problems were only classified as complications according to the CM system if they required medical or surgical treatment. Because the CM system grades complications based on the need for intervention, minor issues managed with routine stoma care, such as sealing paste or dressings, do not necessarily qualify as a surgical complication [10]. Reporting CM-classified complications separately from stoma- and skin-related problems allowed us to distinguish between clinically significant postoperative events and issues related to daily stoma care.

Stoma care in neonates is largely managed by parents at home and can be challenging because of the small abdominal surface and frequent loose stools [17]. Qualitative research has shown that caregivers often experience uncertainty, emotional stress, and a strong need for professional support when caring for their neonate with a stoma [18]. At our institution, families receive structured education from dedicated stoma nurses and have easy access to follow-up and advice through the stoma period. Although caregiver burden was not assessed in this study, close follow-up may contribute to early identification and management of minor problems.

Traditional double-barrel colostomies require two separate stoma sites divided by a skin bridge, whereas the single-site configuration uses one skin opening. Cosmetic outcomes were not formally assessed in this study. However, minimizing abdominal scarring may be relevant in children with ARM, who often undergo multiple surgical procedures during childhood and where long-term body image and psychosocial well-being may be affected [6, 19]. Another approach aiming to reduce visible scarring is the umbilical loop colostomy, in which a double-barreled loop colostomy is created through the umbilicus with a protruding conduit of more than 2 cm above the skin [9]. After closure, only a small scar at the base of the umbilicus remains.

Key strengths of this study are the use of a uniform surgical technique at a single tertiary referral center and detailed documentation of stoma care and skin-related problems during follow-up. Complications were classified according to the CM system, enabling structured and transparent reporting of severity. The limitations include the retrospective design with a risk of incomplete documentation and reporting bias, the absence of a direct comparison group, and a moderate sample size. Patient/parent-reported measures on abdominal scarring, cosmetic outcomes and parental experiences with stoma care are lacking.

## Conclusion

Single-site divided colostomy is a safe and feasible technique in neonates with ARM. Major complications were uncommon, and most stoma- and skin-related problems were minor and manageable. The technique may facilitate stoma care for parents and reduce abdominal scarring.

## Data Availability

No datasets were generated or analysed during the current study.
